# C-shaped canals in first and second mandibular molars from Brazilian individuals: A prevalence study using cone-beam computed tomography

**DOI:** 10.1371/journal.pone.0211948

**Published:** 2019-02-13

**Authors:** Katia R. Vaz de Azevedo, Cristiane B. Lopes, Rosana H. T. L. R. Andrade, Fernanda F. N. Pacheco da Costa, Lúcio S. Gonçalves, Rachel Medeiros dos Santos, Flávio R. F. Alves

**Affiliations:** 1 Department of Endodontics, Faculty of Dentistry, Estácio de Sá University, Rio de Janeiro, RJ, Brazil; 2 Independent researcher, Radiology specialist, Private Oral Radiology Clinic, Rio de Janeiro, RJ, Brazil; University of Brescia, ITALY

## Abstract

**Introduction:**

The study aimed to evaluate, through in vivo tomographic analysis, the prevalence of C-shaped canals in mandibular first and second molars of Brazilian individuals, analyzing its frequency by thirds of the roots, and in contralateral teeth.

**Methods:**

Images of 801 mandibular molars (379 first molars and 422 second molars) from 334 Brazilian individuals (142 men and 192 women) were identified through 1544 cone beam computed tomography (CBCT) exams, obtained from a private oral radiologic clinic. The cross-sectional configurations were analyzed to determine the frequency of C-shaped canals at three different axial levels and classified in categories by three experienced endodontists independently.

**Results:**

The incidence of C-shaped canals was 181 (23%). Considering the type of tooth, 91 (24.01%) were identified in the first molars, and 90 (21.32%) were found in the second molars. The incidence was significantly higher in female individuals (P < 0.05) for both first and second molars. The most common C-shaped canal configurations were: C1 (89.01% for first molars and 90% second molars), followed by C2 (8.79% for first molars and 6.66% for second molars) and C4 (2.19% for the first molars and 3.33% for the second molars). Bilateral C-shaped canals were significantly higher than unilateral for both first and second molars (P < 0.01).

**Conclusions:**

The prevalence of C-shaped canals in mandibular molars of the Brazilian individuals was higher than previously reported for both mandibular first (24.01%) and second molars (21.32%). The incidence was significantly higher in female individuals and the coronal portion of the roots. The classic C-shaped format “C1” was the most frequent anatomical configuration. Furthermore, the prevalence of bilateral C-shaped canals was higher for the first molar (61.70%) and lower for the second molar (38.29%).

## Introduction

The knowledge of root canal anatomy and its variations among the ethnic groups is crucial for adequate coronal access, root canal preparation, and filling, increasing the chances of success of endodontic treatment [1]. One of the most challenges regarding root canal anatomy is the occurrence of C-shaped canals. The most commonly affected teeth are mandibular molars [1–4]. The classic internal morphology of a mandibular molar with C-shaped canals is characterized by slit-like anatomy connecting the mesiolingual, mesiobuccal and distal canals, forming a 180° arc [5]. Roots containing C-shaped canals often have a square or conical configuration [6–8].

The prevalence of C-shaped canals is variable and influenced by the tooth type and the studied population. The highest incidence of C-shaped canals has been reported for the mandibular second molars (3% to 44.6%) [9,10] and the lowest for mandibular first molars (0.16–10%) [11,12]. Differently, the occurrence of C-shaped canals in mandibular third molars was not confirmed in the majority of the studies, except for a study in the North American population that showed a prevalence of 2% [13].

Only two studies evaluated the prevalence of C-shaped canals in the Brazilian population using cone beam computed tomography (CBCT) [14,15]. The first analyzed exams from 154 individuals and found an incidence of 1.7% for the mandibular first molars and 3.5% for mandibular second molars. On the other hand, the second and newest study analyzed exams from 214 patients and found a much higher incidence for the mandibular second molars (15%). The difficulty of the prevalence studies in the Brazilian population is related to its large size. Brazil is the fifth most populated country in the world, and its population is very miscegenated.

When faced with C-shaped canals, endodontists may not visualize the “C” extension, i.e., there is no way to know if it is present along the entire root length or only in the coronal third of the roots. This information is extremely relevant to the clinicians. However, most prevalence studies of C-shaped, including those performed in the Brazilian population, did not analyze the prevalence of C-shaped canals by thirds of the roots. Additionally, no study examined the prevalence of C-shaped canals in contralateral mandibular first molars.

The objective of this study was to evaluate, through in vivo tomographic analysis, the prevalence of C-shaped canals in mandibular first and second molars of Brazilian individuals, analyzing its frequency by thirds of the roots, and in contralateral teeth.

## Materials and methods

### Subjected population

A sample size estimate was calculated considering the Brazilian population size (208.811.000 inhabitants according to the IBGE), a sample error of 5%, a level of confidence of 99%, and a maximum percentage of C-shaped of 15.3% for the mandibular second molars [15] and 1.7% for the mandibular first molar [14]. This calculation was performed using a formula which contains the following parameters: “n”, sample size calculated; “N”, population size; “Z”, normal standard variable related to the confidence level; “p”, true probability of the event; and “e”, sampling error.

$$n=\frac{N.Z^{2}.p.(1-p)}{Z^{2}.p.(1-p)+e^{2}.(N-1)}$$

The sample size needed to estimate the prevalence of C-shaped in the Brazilian population was 344 teeth for mandibular second molars and 45 for mandibular first molars.

After the approval of the research project by the Institutional Research Ethics Committee, images of 510 mandibular first molars and 699 mandibular second molars of 528 Brazilian individuals (209 men and 319 women) were identified in a database with 1544 CBCT exams obtained from a private radiologic clinic in Rio de Janeiro. The exams were carried out from January to April of 2015 using four ICAT Classic units (Imaging Sciences, Hatfield). The images were acquired with voxel size 0.2 mm, 120 kv and FOV limited to 6 cm. The export program of DICOM's files was the XORAN (XoranConnect, Ann Arbor). Before the analysis, the files were identified by a number to guarantee the patients anonymity. Samples were analyzed by three experienced endodontists independently. The examiners were previously gauged to detect C-shaped canals in a set of 100 exams, and the agreement was substantial (Cohen’s Kappa > 0.62). Discordant cases were decided for the most frequent answer. Only the mandibular first and second molars were analyzed. The inclusion criteria were: teeth with no evidence of anterior endodontic treatment and with fully formed roots. Teeth with root resorption, incompletely formed roots, pulp nodule, root canal calcification, metallic crowns, treated canals, or those with difficulty of visualization in the CBCT were excluded when perceived by at least one of the examiners.

The software used in the analysis was the Dental Slice Virtual Navigation version 2014 for Windows (Bioparts Biomedical Prototyping, Brasília, DF, Brazil). Contrast and brightness of the images were modified to ensure the best viewing. The analyses were performed on LCD monitors with a resolution of 1366x768 pixels, in a dark environment. The images from each selected molar were evaluated in axial, coronal and sagittal sections.

The cross-sectional configurations were analyzed to determine the frequency of C-shaped canals at three different axial levels: ‘‘coronal”: 2 mm under the cementoenamel junction, ‘‘apical”: 2 mm above the anatomic apex, and ‘‘middle”: the middle distance between ‘‘coronal” and ‘‘apical”. The C-shaped canals were classified in categories, according to Fan et al. [6]: C1, the shape was an uninterrupted “C” with no separation or division; C2, the canal shape resembled a semicolumn resulting from a discontinuation of the “C” outline; C3, two or three separate canals and both angles; C4, only one round or oval canal in that cross-section; and C5, no canal lumen could be observed.

### Statistical analysis

Fisher's exact test was used to analyze the influence of gender and tooth type on C-shaped canals prevalence. The same test was also used to compare the prevalence of bilateral (contralateral teeth) and unilateral C-shaped canals. The level of significance was set at 5% (P <0.05).

## Results

The interobserver reliability was high for all evaluated teeth regarding C-shaped canals identification (Cohen’s Kappa > 0.92) and their classification (100% of agreement).

The selected sample included 801 mandibular molars (379 mandibular first molars and 422 mandibular second molars) from 334 patients (142 males and 192 females) with age varying from 19 to 95 years.

The frequency of C-shaped canals was 181 (22.59%). Ninety-one (24.01%) were identified in the first molars (64 from females patients and 27 from males) and 90 (21.32%) were found in the second molars (62 from females patients and 28 from males). The difference between the genders was statistically significant (P < 0.05) for both the first and second molars.

**Fig 1:**
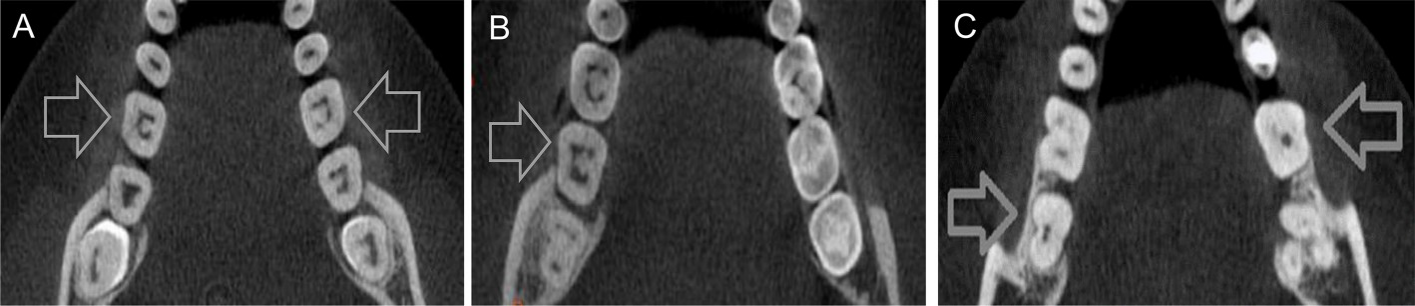
A, Image of bilateral class 1 C-shaped canals on mandibular first molars. Note the uninterrupted “C” form line. B, Image of unilateral class 2 C-shaped canals on a mandibular second molar. Note the discontinuation of the “C” outline. C, Image of bilateral class 4 C-shaped canals on mandibular second molars. Note the presence of only one round canal in the cross-section.

The most frequent C-Shaped canal configurations was C1 (81/91 for the first molars, 89.01% and 81/90 for the second molars, 90%) followed by C2 (8/91 for the first molars, 8.79% and 6/90 on for the second molars, 6.66%) and C4 (2/91 for the first molars, 2.19% and 3/90 for the second molars, 3.33%) (Fig 1). The other types were not identified in any tooth (Table 1).

**Table 1:** Frequency of the different types of C-Shaped configurations.

Tooth	C1	C2	C4	Total
36	44	2	0	46
37	37	6	2	45
46	41	2	0	43
47	40	4	3	47
**Total**	162	14	5	181

Overall, C-shaped canals were much more frequent in the coronal third of the roots than in the middle and apical thirds for both first (89%) and second molars (88%) (p<0.05). Sixteen teeth presented the C-shaped configuration in at least two-thirds of the roots. Only three teeth presented C-shaped configuration in all the three-thirds of the roots (Table 2).

**Table 2:** Frequency of the C-Shaped canals per third of the roots.

Tooth	Coronal	Middle	Apical	Coronal and middle	Coronal and apical	Middle and apical	Coronal, Middle and apical
36	40	6	0	0	0	0	0
37	41	5	0	1	1	1	1
46	38	7	1	2	0	0	0
47	41	10	2	4	2	2	2

A total of 263 pairs of contralateral teeth was identified among the evaluated CBCT exams, 99 of first molar and 164 of the second molar. Among them, 47 pairs presented C-shaped canals in both teeth, 29 of first molars (61.70%) and 18 of second molars (38.29%). On the other hand, some pairs presented unilateral C-shaped canals, 14 in the first molars (29.78%) and 27 in the second molars (57.44%). The frequency of bilateral C-shaped canals was significantly higher when compared with unilateral, for both mandibular first and second molars (P<0.01).

## Discussion

The present study evaluated the prevalence of C-shaped canals in first and second mandibular molars from Brazilian individuals. CBCT images from 801 teeth were carefully analyzed by three experienced endodontists to determine the frequency of this occurrence by thirds of the roots and in contralateral teeth. The use of CBCT has been indicated for the diagnosis of root canal aberrations [16] including C-Shaped canals [17,18]. However, before indicating CBCT exams, it is necessary to follow scientifically based recommendations [19] and, when correctly indicated, to guarantee that the dose is kept “*As Low as Reasonably Achievable*” [20]. In the present study, the authors did not participate in the CBCT indications. All tomographic exams were acquired using a 0.2-mm voxel size, which in previous studies, was demonstrated suitable for C-shaped canals detection [14,21,22].

The sample size used in the present study was larger compared to previous studies in Brazilian population using CBCT [14,15] or micro-computed tomography [23]. Sample size calculation is critical during the planning of scientific studies. Insufficient or small sample size may impair the estimation of the frequency of a clinical condition. Contrarily, an increase in the sample size reduces the error. It is important to emphasize that the present study was the only one in which a sample size calculation was performed to estimate the prevalence of C-shaped canals in teeth in Brazilian individuals.

Studies in other countries showed a percentage of C-shaped canals varying from 0.16% [11] to 10% [12] for the mandibular first molar and from 1.9% [9] to 45% [10] for the second mandibular molar. On the other hand, the few available studies in the Brazilian population demonstrated that the prevalence of C-shaped canals could vary from 3.5% to 33% [14,15,23] for the second mandibular molar. As for the first mandibular molar, the reported prevalence was 1.7% [14]. Compared with CBCT studies [14,15], the present one found a very higher prevalence of C-shaped canals for the mandibular first molars (24%). Nonetheless, the prevalence for the second molars (21%) was similar to previous studies not only performed in the Brazilian population [15] but also in other populations [24,25]. Otherstudies found much higher percentages compared with the present one for the second molar [10,26,27], including one performed in the Brazilian population using microcomputed tomography [23]. Differences in the evaluation method or the sample size can help to understand these differences. Additionally, comparing the present results concerning the second molar (21% of prevalence) with data from a previous microcomputed tomography study performed in the Brazilian population (33% of prevalence) it could be noted that the analysis of the present study was conducted with a high level of accuracy.

The clinical relevance of the present findings is incontestable since C-shaped canals are considered one of the most anatomical challenges in Endodontics. The complex anatomy of these canals, characterized by the presence of narrow and irregular areas can act as a reservoir of soft-tissues, microorganisms, and dentinal debris that might not be removed entirely, impairing the success of treatment. The detection of C-shaped canals by the clinicians is not as easy as expected. Although C-shaped canals must be suspected when the molar roots seem to be fused, the radiographic appearance of two distinct roots does not preclude the existence of C-shaped canals [7]. The use of an operating microscope is highly recommendable when the clinician suspects the presence of C-shaped canals since the magnification increases detection of additional canals [28,29]. Besides, the microscope could help to prevent perforations in these cases.

Another interesting finding from the present study was the high prevalence of bilateral C-shaped canals in the first molars (62%). To the best of our knowledge, there is no study evaluating the frequency of C-shaped canals between contralateral mandibular first molars. On the other hand, bilateral C-shaped canals in second molars were not much frequent (38%). A similar prevalence was found in another Brazilian study (32%) [15]. Depending on the population, this prevalence can be much lower as verified in Turkish individuals (6.3%) [22] or much higher as observed in Koreans (82%) [30].

Regarding the configuration type, the classic C-shaped format “C1” was the most common type, detected in 89% and 90% of the mandibular first and second molars respectively. Contrarily, the types C2 and C3 were identified in a few cases. This result is in accordance with the classic study of Fan et al. [6] that found a prevalence of 89% on mandibular second molars. The types C2 and C4 were found in few teeth in that study. Another similar result from both studies was that the coronal third was the most frequent location of C-shaped canals. These results claim the use of instruments specially designed for operation in oval or flattened root canals during the preparation of C-shaped canals. A study compared the Self-Adjusting File, which adapts itself to the root canal anatomy and found that this instrument was more effective in shaping the C-shaped canal walls compared with a conventional rotary system (ProTaper) [31].

## Conclusions

The prevalence of C-shaped canals in mandibular molars of the Brazilian individuals was higher than previously reported for both first (24%) and second molars (21%). The incidence was significantly higher in female individuals and the coronal portion of the roots. The classic C-shaped format “C1” was the most frequent anatomical configuration. Furthermore, the prevalence of bilateral C-shaped canals was higher for the first molar (62%) and lower for the second molar (38%). These findings reinforce the need for advanced technologies including the operating microscope and instruments specially designed for the preparation of oval or flattened canals, for the treatment of C-shaped canals. Additionally, CBCT is a valuable resource in the clinical study and treatment planning of this complex root canal anatomy.
